# Coptisine Attenuates Diabetes—Associated Endothelial Dysfunction through Inhibition of Endoplasmic Reticulum Stress and Oxidative Stress

**DOI:** 10.3390/molecules26144210

**Published:** 2021-07-11

**Authors:** Yan Zhou, Chunxiu Zhou, Xutao Zhang, Chi Teng Vong, Yitao Wang, Wai San Cheang

**Affiliations:** State Key Laboratory of Quality Research in Chinese Medicine, Institute of Chinese Medical Sciences, University of Macau, Macao SAR 999078, China; yc07517@umac.mo (Y.Z.); mc05835@um.edu.mo (C.Z.); mb95825@um.edu.mo (X.Z.); gigictvong@um.edu.mo (C.T.V.); ytwang@um.edu.mo (Y.W.)

**Keywords:** coptisine, diabetes, endoplasmic reticulum stress, endothelial dysfunction, oxidative stress

## Abstract

Coptisine is the major bioactive protoberberine alkaloid found in *Rhizoma Coptidis.* Coptisine reduces inflammatory responses and improves glucose tolerance; nevertheless, whether coptisine has vasoprotective effect in diabetes is not fully characterized. Conduit arteries including aortas and carotid arteries were obtained from male C57BL/6J mice for *ex vivo* treatment with risk factors (high glucose or tunicamycin) and coptisine. Some arterial rings were obtained from diabetic mice, which were induced by high-fat diet (45% kcal% fat) feeding for 6 weeks combined with a low-dose intraperitoneal injection of streptozotocin (120 mg/kg). Functional studies showed that coptisine protected endothelium-dependent relaxation in aortas against risk factors and from diabetic mice. Coptisine increased phosphorylations of AMPK and eNOS and downregulated the endoplasmic reticulum (ER) stress markers as determined by Western blotting. Coptisine elevates NO bioavailability and decreases reactive oxygen species level. The results indicate that coptisine improves vascular function in diabetes through suppression of ER stress and oxidative stress, implying the therapeutic potential of coptisine to treat diabetic vasculopathy.

## 1. Background

Cardiovascular disease (CVD) is a multifactorial disease with a high mortality rate in the world, where obesity and diabetes are the major risk factors [1]. Diabetes is a metabolic disease characterized by insulin resistance or deficiency [2]. Studies have shown that hyperglycemia causes endothelial damage and thereby endothelial dysfunction, which is the main serious complication of diabetes [3]. The recognized complex mechanisms by which hyperglycemia modifies endothelial function include reduction in nitric oxide (NO) bioavailability [4], production of vasoconstrictors such as endothelin-1 (ET-1) [5], increased generation of reactive oxygen species (ROS) [6], and glycation of proteins and lipids [7]. NO bioavailability is an important index for determining endothelial function. Any situation in which the endothelial nitric oxide synthase (eNOS) activity is reduced or the ROS production is elevated can lead to a lowered NO bioavailability and, consequently, impair endothelium-dependent vasodilatations. The phosphorylation of eNOS is decreased in diabetic mouse aortas and AMP-activated protein kinase (AMPK) phosphorylation is one of the major types of signalling to stimulate eNOS [8,9].

Endoplasmic reticulum (ER) is a crucial organelle in which protein synthesis, maturation, folding and trafficking take place. Only properly folded proteins can be destined to cellular organelles or cell surface; nevertheless, misfolded or unfolded proteins are retained in the ER to be eventually degraded [10]. Disruption of the aforementioned processes results in accumulation of newly synthesized unfolded proteins in the ER and this condition is referred to as ER stress [11]. In response to ER stress, the three branches of the unfolded protein response (UPR) are activated, initiated by three ER membrane-associated proteins—PKR-like endoplasmic reticulum kinase (PERK), inositol requiring enzyme 1 (IRE1), and activating transcription factor 6 (ATF6)—and the engagement of complex downstream signalling pathways [11,12]. The transcriptional factors downstream of these proteins collectively induce unfolded protein response target genes involved in protein synthesis, oxidative stress, inflammation, and apoptosis [8]. Of note, excessive nutrients such as hyperglycemia related to metabolic diseases can induce ER stress [13].

**Figure 1:**
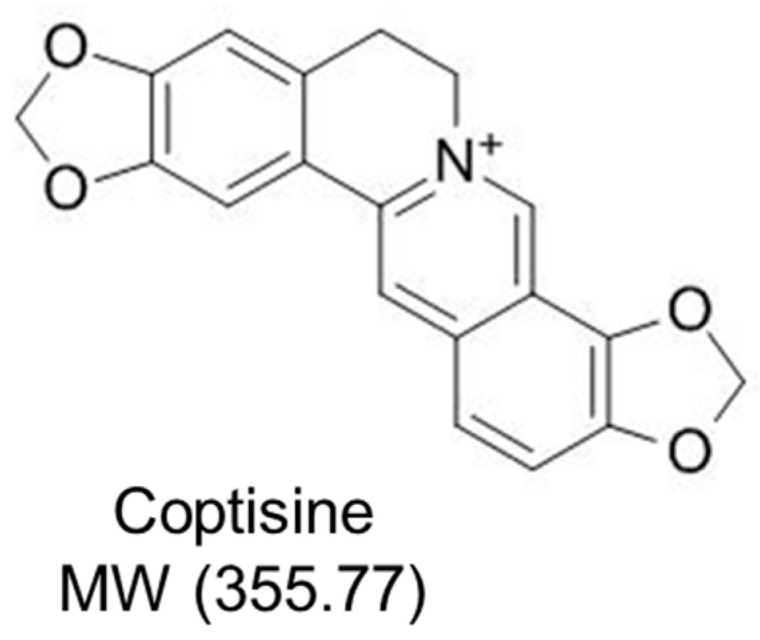
Structure of coptisine.

*Rhizoma Coptidis* (RC) is the dried rhizome of Ranunculaceae plants, which is a common and famous Chinese medicine used for more than two thousand years [14]. In addition to the traditional antibacterial effects, RC shows a wide range of biological activities, such as anti-cancer, anti-inflammatory and other activities [15,16]. In recent years, studies have found that RC exerts beneficial effects against the main risk factors of CVD [17], including anti-hyperglycemia and anti-diabetic effects [18]. These characteristics are attributed to the main active compositions, protoberberine alkaloids in RC, which include coptisine (Figure 1), jatrorrhizine, berberine, epiberberine and palmatine [19]. The present study aims to evaluate whether coptisine protects against endothelial dysfunction in diabetes and whether the AMPK/eNOS pathway, ER stress or oxidative stress is involved.

## 2. Results 

### 2.1. Coptisine Ameliorates Endothelial Dysfunction Associated with Diabetes

**Figure 2:**
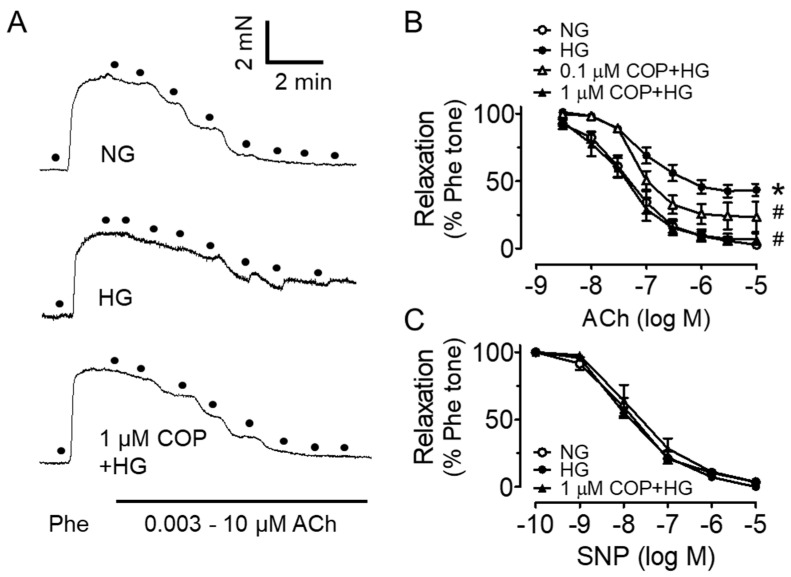
Vasoprotective effect of coptisine (COP) in aortas from C57BL/J mice ex vivo. (**A**) Representative traces and (**B**) summarized data showing that high glucose (HG, 30 mM, 48 h) impaired acetylcholine (ACh)-induced endothelium-dependent relaxations as compared to the control (normal glucose, NG; 5.55 mM glucose in DMEM with addition of mannitol as osmotic control), and that COP improved the relaxations. (**C**) Sodium nitroprusside (SNP)-induced endothelium-independent relaxations were not affected. Results are the mean ± SEM of 3 experiments. * *p* < 0.05 vs. NG; # *p* < 0.05 vs. HG.

**Figure 3:**
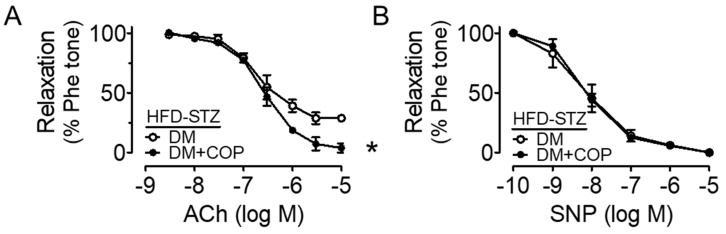
Coptisine (COP) protects endothelial function in diabetic mice. (**A**) Diabetic mouse model was induced by high fat diet (45% kcal% fat) and i.p. injection of streptozotocin (120 mg/kg) (HFD-STZ) and ex vivo treatment of COP (1 µM, 16 h) improved Ach-induced relaxations in diabetic (DM) mouse aortas. (**B**) SNP-induced relaxations were unaffected. Results are the mean ± SEM of 3 experiments. * *p* < 0.05 vs. DM.

Mouse aortas were incubated with high glucose (30 mM, 48 h) to mimic the hyperglycemic condition in diabetes and we found that ex vivo high glucose exposure impaired the Ach-induced EDRs when compared to the control (5.55 mM glucose present in DMEM with addition of mannitol as osmotic control), and such impairments were reversed by co-treatment of COP in a dose-dependent manner (Figure 2A,B, and Table 1). COP at 0.1 µM moderately improved EDRs, while a higher concentration at 1 µM was more effective in producing EDRs that were comparable to the normal glucose control (NG, mannitol used as an osmotic control). Besides, endothelium-independent relaxations to SNP were similar among the four groups (Figure 2C), which shows that the response of vascular smooth muscle to NO was not affected. Importantly, diabetes was induced in mice by high-fat diet (45% kcal% fat) together with single intraperitoneal injection of streptozotocin (120 mg/kg) and was confirmed by the 12 h fasting blood glucose: 11.3 mM, 14.5 mM and 12.2 mM for three different mice. The impaired EDRs in aortas from these diabetic (DM) mice were enhanced by coptisine treatment (1 µM, 16 h) (Figure 3A) whilst SNP-induced endothelium-independent relaxations remained unaltered (Figure 3B). 

### 2.2. Coptisine Increases eNOS Phosphorylation and NO Bioavailability

**Figure 4:**
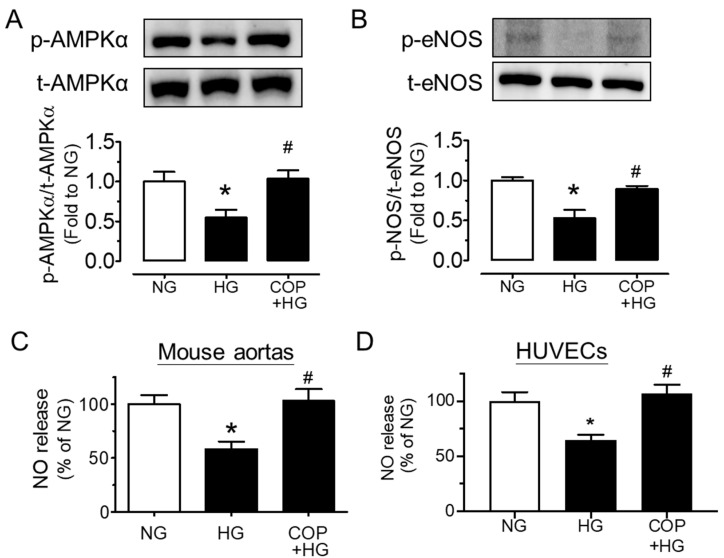
Coptisine (COP) stimulates AMPK/eNOS pathway and NO production. Representative blots and summarized data showing (**A**) the phosphorylation of AMPKα at Thr172 (p-AMPKα; 62 kDa) and (**B**) phosphorylation of eNOS at Ser1177 (p-eNOS; 140 kDa) as compared to their corresponding total protein in mouse aortas treated with high glucose (HG, 30 mM) and COP (1 µM) for 48 h. NO release from (**C**) mouse aortas and (**D**) HUVECs upon high glucose stimulation and co-treatment with COP (1 µM) as assessed by measuring the nitrite level in culture medium. Results are the mean ± SEM of 3 experiments. * *p* < 0.05 vs. NG; # *p* < 0.05 vs. HG.

For the underlying mechanism of the vasoprotective effect of coptisine, we examined whether the AMPK/eNOS pathway is involved. Upon stimulation to high glucose, the phosphorylations of AMPKα at Thr172 and eNOS at Ser1177 (indicators of NO bioavailability) in mouse aortas were significantly reduced and were normalized by co-incubation of COP (1 µM), whereas the total protein levels of AMPKα and eNOS were unchanged (Figure 4A,B). Moreover, the nitrite level in the conditioned culture medium was suppressed by high glucose exposure and was remarkably elevated by COP treatment in both mouse aortas (Figure 4C) and HUVECs (Figure 4D), indicating an improved NO bioavailability.

### 2.3. Coptisine Suppresses ER Stress and Oxidative Stress

**Figure 5:**
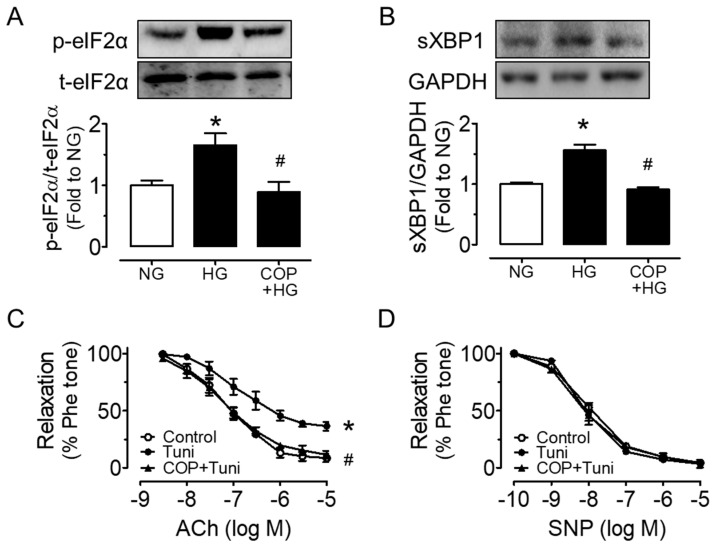
Coptisine (COP) inhibits ER stress. Representative blots and summarized data showing (**A**) the phosphorylation of eIF2α at Ser52 (p-eIF2α; 36 kDa) compared to its total protein (t-eIF2α) and (**B**) spliced XBP1 (sXBP1; 54 kDa) compared to GAPDH (36 kDa) in mouse aortas treated with high glucose (HG, 30 mM) and COP (1 µM) for 48 h. (**C**) ER stress inducer tunicamycin (tuni; 2 µg/ml, 24 h) impaired the ACh-induced endothelium-dependent relaxations in aortas from C57BL/J mice ex vivo, which were improved by COP (1 µM), with no effect on (**D**) SNP-induced relaxations. Results are the mean ± SEM of 3 experiments. * *p* < 0.05 vs. NG or Control; # *p* < 0.05 vs. HG or Tuni.

**Figure 6:**
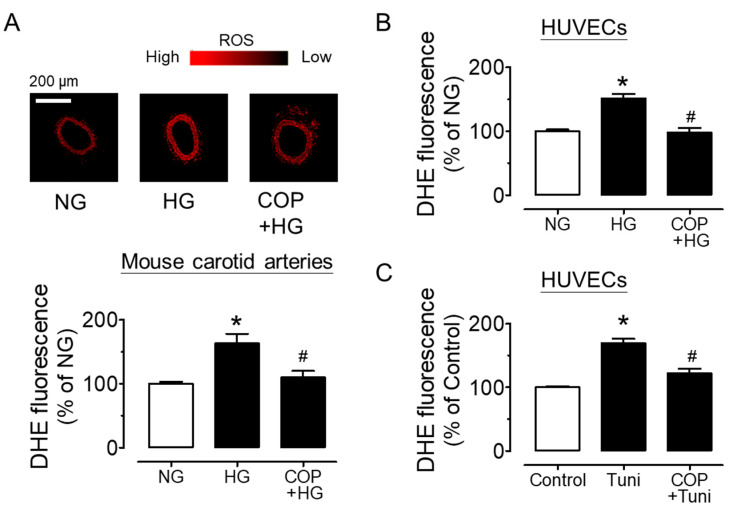
Coptisine (COP) reduces oxidative stress. Exposure to high glucose (30 mM, 4h) increased the level of reactive oxygen species (ROS) in (**A**) mouse carotid arteries and (**B**) HUVECs, and such elevation was decreased by COP at 1 µM, as measured by dihydroethidium (DHE) staining. (**C**) COP treatment (1 µM) suppressed the tunicamycin (tuni; 2 µg/mL, 1 h)-induced ROS generation in HUVECs. Results are the mean ± SEM of 4 experiments. * *p* < 0.05 vs. NG or Control; # *p* < 0.05 vs. HG or Tuni.

COP reduced the high glucose-induced upregulations of ER stress markers including phosphorylations of eIF2α at Ser52 and spliced XBP1 in mouse aortas (sXBP1) (Figure 5A,B). Since high glucose triggered ER stress, we next verified the impact of ER stress on vascular function by examining EDRs in the presence of ER stress inducer tunicamycin (2 µg/mL, 24 h). The direct induction of ER stress on blood vessels by tunicamycin resulted in impaired EDRs, which was effectively attenuated by co-treatment with 1 µM COP (Figure 5C), and SNP-induced endothelium-independent relaxations were not affected (Figure 5D). In addition, COP reduced high glucose (30 mM, 4 h)-induced oxidative stress in mouse carotid arteries (Figure 6A) as well as in HUVECs (Figure 6B), as measured by DHE fluorescence. The induction of ER stress by tunicamycin (2 µg/mL, 1 h) also increased ROS generation, which was reversed by COP in HUVECs (Figure 6C). The time-dependent effect of high glucose and tunicamycin on ROS levels was determined and the results showed that the induction of ROS was the highest, at 4 h, for high glucose, and was 1 h for tunicamycin exposure (data not shown); thus, these two time points were selected for accessing the anti-oxidative effect of COP.

## 3. Methods

### 3.1. Animals 

The use of animals and research protocol were in accordance with the National Institutes of Health guidelines for the Care of Use of Laboratory Animals, and approved by the Animal Research Ethics Committee, University of Macau, Macao SAR, China (reference No: UMARE-028-2020). Male C57BL/6J mice were supplied by the Faculty of Healthy Science Animal Centre of University of Macau and housed in a temperature-controlled room (22–24 °C) with a 12 h light/dark cycle. The mice were fed with standard chow diet and sacrificed at the age of 10–12 weeks to obtain the thoracic aortas and carotid arteries for ex vivo treatment: six mice were used for functional studies and three mice for Western blotting. Another three mice, at the age of 6 weeks, were fed with high fat diet (45% kcal% fat; Shuyishuer Bio, Changzhou, China) for 6 weeks combined with a low-dose intraperitoneal injection of streptozotocin (120 mg/kg) to establish a type 2 diabetic model. The fasting blood glucose (12 h fasting) was determined using a commercial blood glucose meter and the mice with fasting blood glucose of >11 mM were considered as diabetic. 

### 3.2. Ex Vivo Culture of Mouse Aortas

After mice were sacrificed, mouse thoracic aortas and carotid arteries were dissected in sterile PBS and then incubated in DMEM supplemented with 10% FBS and 1% penicillin/streptomycin (Gibco, Gaithersbury, MD, USA). High glucose (HG; 30 mM, 48 h; Sigma-Aldrich, St. Louis, MO, USA) and tunicamycin (tuni; 2 µg/ml, 24 h; Sigma-Aldrich) were added individually into the culture medium that bathed aortic rings in a humidified atmosphere of 5% CO_2_ at 37 °C. Some arterial rings were co-treated with coptisine (COP, 0.1 or 1 µM) which was purchased from Shanghai Aladdin Bio-Chem Technology Co. Ltd. (Shanghai, China) (purity > 98%). After the incubation period, segments were transferred to fresh Krebs solution for functional studies in a wire myograph and were frozen for Western blotting and fluorescence imaging. 

### 3.3. Isometric Force Measurement in Wire Myograph

Segments of mouse aortas (~2 mm) were mounted to a Multi Myograph System (Danish Myo Technology, Aarhus, Denmark) and changes in isometric tension were measured as previously described [8,9]. Mouse aortas were stretched to an optimal baseline tension of 3 mN and were then allowed to equilibrate for 60 min before the start of the experiment. Each ring was first contracted by 60 mM KCl and rinsed several times in Krebs solution. To examine endothelium-dependent relaxations (EDR), after washout, phenylephrine (Phe, 1 μM, α1-adrenoceptor agonist, Sigma-Aldrich) was used to produce a steady contraction and subsequently relaxed by cumulative addition of acetylcholine (Ach, 3 nM–10 µM, muscarinic acetylcholine receptor agonist, Sigma-Aldrich). Endothelium-independent relaxations were also measured in response to sodium nitroprusside (SNP, 1 nM–10 µM, exogenous NO donor, Sigma-Aldrich). Each experiment was performed on rings prepared from different mice.

### 3.4. Western Blotting

Aortas were snap frozen in liquid nitrogen and subsequently homogenized in ice-cold RIPA lysis buffer (Beyotime Biotechnology, Shanghai, China). The lysates were placed on ice for 30 min and then centrifuged for 20 min at 20,000g. The supernatant was collected and measured for protein concentration by BCA assay (Beyotime). Each protein sample (20 µg) was electrophoresed through 10% sodium dodecyl sulfate polyacrylamide gel electrophoresis (SDS-PAGE) and transferred to PVDF membrane (Millipore, Billerica, MA, USA) using wet transfer (BIO-RAD, Hercules, CA, USA) to detect the protein expression (both phosphorylated and total proteins) of AMPK, eNOS and ER stress markers. All primary antibodies were purchased from Cell Signaling Technology (Danvers, MA, USA). GAPDH was selected as a housekeeping protein for checking the equal loading of each sample. Protein bands were visualized with an American ECLTM Advanced Western Blotting Detection Kit (GE Healthcare Life Sciences, Uppsala, Sweden) and scanned using the ChemiDocTM MP Imaging System (BIO-RAD).

### 3.5. Culture of Human Umbilical Cord Vein Endothelial Cells (HUVECs)

HUVECs obtained from Lonza were grown in EGM supplemented with Bulletkit (Lonza, Verviers, Belgium). Cells were grown in 75 cm^2^ flasks and maintained at 37 ℃ in a 95% humidified air/5% CO_2_ atmosphere. Medium was changed every two days. Confluent cells were passaged by trypsinization (0.25% trypsin with 2.5 mM EDTA in PBS). Experiments were performed on cells at passage 4–8 for treatment with high glucose (30 mM, 48 h) or tunicamycin (2 µg/mL, 24 h), and for co-treatment with coptisine (1 µM) when 80–90% confluency was achieved.

### 3.6. Detection of Intracellular Oxidant Formation by Dihydroethidium (DHE) Fluorescence

The intracellular ROS level was determined by DHE (Invitrogen, Eugene, OR, USA) as previously described [8]. Isolated carotid arterial rings, after ex vivo treatments, were frozen in OCT compound (Tissue-Tek, Tokyo, Japan) and sliced into sections of 10-μm thickness using a cryostat. The frozen sections of arterial rings or treated HUVECs were incubated in DHE (5 μM)-containing normal physiological saline solution (NPSS) in dark at 37 °C for 15 min where NPSS contained (mM): 140 NaCl, 5 KCl, 1 CaCl2, 1 MgCl2, 10 glucose, and 5 HEPES (pH 7.4). Fluorescence images were obtained using the Leica TCS SP8 Confocal Laser Scanning Microscope System (Leica Microsystems, Wetzlar, Germany) at 515 nm excitation and 585 nm emission. 

### 3.7. Determination of NO Generation

Aortic segments or HUVECs were treated with different drugs and the culture medium was collected to determine NO levels by a colorimetric assay kit involved the Griess reaction (Sigma-Aldrich) according to manufacturer’s instructions. Absorbance was read at 548 nm using the SpectraMax M5 microplate reader (Molecular Devices, Silicon Valley, CA, USA). Protein content was measured on cell lysate by the BCA assay and used to normalize the nitrite values.

### 3.8. Statistical Analysis

All data are showed as mean ± standard error of mean (SEM) of n independent experiments. Relaxation in each aortic segment is expressed as the percentage of the contraction induced by phenylephrine. The negative logarithm of the dilator concentration that caused 50% of the maximum response (*p*D_2_) and the maximum relaxation (*E*_max_%) were calculated. Student’s *t*-test or one-way ANOVA, followed by Bonferroni post hoc tests for more than two treatments, was applied. *p* < 0.05 is considered to be statistically significant.

## 4. Discussion

The present results suggest that coptisine has sufficient therapeutic value to prevent endothelial dysfunction in diabetes; this probably occurs through the inhibition of ER stress and oxidative stress, as well as the restoration of NO production in arteries, based on the following observations. First, ex vivo treatment with COP reversed the impairment of EDRs induced by high glucose and in aortas from diabetic mice. Second, COP upregulated the phosphorylations of AMPKα at Thr172 and eNOS at Ser1177 to restore NO availability in mouse aortas and HUVECs. Third, COP treatment suppressed ER stress markers and prevented the impairment of EDRs induced by ER stress inducer tunicamycin. Lastly, COP inhibited the high glucose-induced oxidative stress in mouse aortas as well as HUVECs and showed similar inhibitory effect against ROS generation triggered by tunicamycin. 

COP is the major bioactive compound in RC and was found to affect vascular functions. COP exerts a vasorelaxant effect, which is both endothelium-dependent and endothelium-independent, at high concentrations in rat aortas [20]. Furthermore, COP shows anti-proliferative activity in rat vascular smooth muscle cells [21]. Chronic inflammation contributes to the progression and development of vascular dysfunction associated with metabolic disorders. A previous study demonstrated that COP exerts anti-inflammatory activity [22]. A recent study demonstrated that COP ameliorates renal injury and vascular endothelial function in systemic lupus erythaematosus [23]. On the other hand, COP improves glucose metabolism in diabetic mice through AMPK activation [24]. Here, we found that COP at low concentrations 0.1 µM and 1 µM was effective in enhancing endothelium-dependent relaxations in conduit arteries under hyperglycemia condition or from diabetic mice.

Phosphorylation/activation of AMPK is one of the major signals to stimulate eNOS activity [25]. Additionally, AMPK activation exerts an inhibitory effect on ROS production associated with diabetes [26,27]. Our previous findings also support that AMPK is physiological suppressor of ER stress and that the modulation of ER stress is crucial for vascular function [8,9]. ER stress can also trigger ROS generation via NADPH oxidases, mainly Nox2 and Nox4 [28]. In line with the previous evidence, we found that COP increased AMPK and eNOS activities, accompanied by enhanced NO bioavailability. COP also downregulated the ER stress markers, such as phosphorylation of eIF2α downstream of the PERK pathway and splicing of XBP1 downstream of the IRE1 pathway. We then showed that ER stress induction by tunicamycin caused endothelial dysfunction and elevated ROS generation, which were reversed by COP. Of note, eNOS activity is reduced by ER-stress-induced apoptosis and inflammation [29], leading to lowered NO bioavailability and vascular dysfunction [30,31,32]. Collectively, our data suggest that COP restores vascular homeostasis in diabetes. Future in vivo and clinical investigations are required to prove the vascular benefits of coptisine in diabetes. 

## 5. Conclusions

The current study provides novel findings supporting the vascular beneficial effects of coptisine to protect against diabetes-associated endothelial dysfunction, largely mediated through activation of the AMPK/eNOS pathway to increase NO production, as well as the alleviation of ER stress and oxidative stress. These results strengthen the prospect of the potential use of coptisine as a therapeutic agent or healthcare supplement for combating diabetic vasculopathy.

## Figures and Tables

**Table 1:** *p*D_2_ and *E*_max_ (%) values for acetylcholine (ACh)-induced relaxations of mouse aortas exposed to high glucose (HG) and coptisine (COP) with mannitol as control (NG). Results are the mean ± SEM of 3 experiments. * *p* < 0.05 vs. NG; # *p* < 0.05 vs. HG.

Treatment	*p*D_2_	*E*_max_ (%)
NG	7.32 ± 0.10	96.23 ± 2.80
HG	7.08 ± 0.13	58.26 ± 2.70 *
0.1 µM COP + HG	7.18 ± 0.15	79.49 ± 4.12 #
1 µM COP + HG	7.45 ± 0.12	93.92 ± 3.12 #

## Data Availability

The data presented in this study are available on request from the corresponding author.

## Sample Availability

Samples of the Western blotting (protein) are available from the authors.
